# Dual functional Phi29 DNA polymerase-triggered exponential rolling circle amplification for sequence-specific detection of target DNA embedded in long-stranded genomic DNA

**DOI:** 10.1038/s41598-017-06594-1

**Published:** 2017-07-24

**Authors:** Xiao-Yu Li, Yi-Chen Du, Yu-Peng Zhang, De-Ming Kong

**Affiliations:** 1 State Key Laboratory of Medicinal Chemical Biology, Nankai University, Tianjin, 300071 P.R. China; 2 Tianjin Key Laboratory of Biosensing and Molecular Recognition, Research Center for Analytical Sciences, Nankai University, Tianjin, 300071 P.R. China; 3 Collaborative Innovation Center of Chemical Science and Engineering (Tianjin), Tianjin, 300071 P.R. China

## Abstract

An exonucleolytic digestion-assisted exponential rolling circle amplification (RCA) strategy was developed for sensitive and sequence-specific detection of target DNA embedded in long-stranded genomic DNA. Herein, Phi29 DNA polymerase plays two important roles as exonuclease and polymerase. Long-stranded genomic DNAs can be broken into small DNA fragments after ultrasonication. The fragments that contain target DNA, hybridize with a linear padlock probe to trigger the formation of a circular RCA template. The tails protruding from the 3′-end of the target DNA sequences are then digested by the 3′ → 5′ exonuclease activity of Phi29 DNA polymerase even if they fold into a double-stranded structure. The digested DNA fragments can then initiate subsequent RCA reaction. RCA products, which are designed to fold into G-quadruplex structures, exponentially accumulate when appropriate nicking endonuclease recognition sites are introduced rationally into the RCA template. This method is demonstrated to work well for real genomic DNA detection using human pathogen *Cryptococcus neoformans* as a model. In addition, this work has two other important discoveries: First, the presence of a 3′-tail can protect the RCA primer from degradation by Phi29 DNA polymerase. Second, 3′ → 5′ exonucleolytic activity of Phi29 DNA polymerase can work for both single- and double-stranded DNA.

## Introduction

Sequence-specific detection of target DNA using reliable, cost-effective and sensitive strategies often attracts broad attention due to its importance in clinical diagnosis and genetic research. In the past few decades, significant and substantial progress has been made in this field^1–5^. In order to realize ultrasensitive detection of target DNA, many promising methods have been designed on the basis of various DNA amplification techniques, including polymerase chain reaction (PCR)^6, 7^, rolling circle amplification (RCA)^8–10^, exonuclease III-aided target recycling^11, 12^, strand-displacement signal amplification^13–16^, hybridization chain reaction^17, 18^ and nicking endonuclease signal amplification^19, 20^. Of all these DNA amplification techniques, PCR is the most well-known and frequently used. However, PCR has some intrinsic limitations, including the requirement of rapid thermal cycling, precise temperature control, thermostable DNA polymerases and high denaturation temperature. This makes it difficult to execute point-of-service analyses. To overcome these, some isothermal DNA amplification techniques^21^, which can be performed at a constant and relatively low temperature, have been designed and widely applied in DNA-sensing applications.

As an important isothermal DNA amplification technique, RCA has attracted more and more attention in biosensing applications and various RCA-based DNA detection strategies have been reported^19, 22–32^. However, most of these works are restricted to the detection of artificial synthetic short-stranded DNA oligonucleotides^19, 27–32^. These short-stranded target DNA might be used as indicators for the genomic DNA of interest (e.g. pathogen genes, oncogenes, or antioncogenes) because the possibility of identical DNA sequence occurring in other non-target genomes is only 1/4^n^ (n is the nucleotide number in the short-stranded target DNA). In practical applications, however, the target DNA is often embedded in long-stranded genomic sequences. There are two ways to cut long-stranded genomic DNA into smaller fragments. One is nuclease-mediated biochemical cleavage. Although site-specific, this method requires the occurrence of nuclease recognition sites near the target DNA sequence, thus the scope of its application might be restricted. The other way is mechanical breakage via ultrasonication. This method has no particular requirements for the DNA sequence and can work for all the genomic DNA of interest, but the main difficulty is that the target DNA may be embedded in DNA fragments with random lengths.

RCA-based DNA sensors mainly work in three ways: (1) Target DNA is used to trigger the formation of the circular RCA template, and an additional RCA primer is needed^19, 28^. (2) Target DNA is used as the RCA primer, and preformed circular RCA template is needed^29, 30^. (3) Target DNA plays both roles^31, 32^. Compared to the first two ways, the last one might provide relatively easier experimental operation, simpler reaction components and thus lower background signal. However, such a strategy has never been combined with ultrasonication to detect target DNA embedded in long-stranded genomic DNA. One reason might be that researchers take for granted that the 3′-ends of the DNA fragments should fully match the RCA template when they are used as the primer to initiate the RCA reaction, but ignore the 3′ → 5′ exonuclease activity of Phi29 DNA polymerase^33–40^.

Herein, we demonstrate that the 3′ → 5′ exonuclease activity of Phi29 DNA polymerase can digest the tail protruding from the 3′-end of target DNA even if the 3′-tail folds into an intramolecular double-stranded structure, thus it can efficiently convert different lengths of target DNA-containing fragments into RCA primers. Based on this, an easy-to-operate biosensing strategy was designed and was demonstrated to work well for the sensitive and sequence-specific detection of target DNA embedded in long-stranded genomic DNA using the human pathogen *Cryptococcus neoformans* (*C*. *neoformans*) as a model. Target DNA-triggered production of circular RCA template confers that the method has extraordinarily high detection specificity, and can easily discriminate single-nucleotide polymorphisms (SNPs).

## Results and Discussion

### Detection of target DNA embedded in DNA fragments by exponential exonucleolytic digestion-assisted RCA

**Figure 1:**
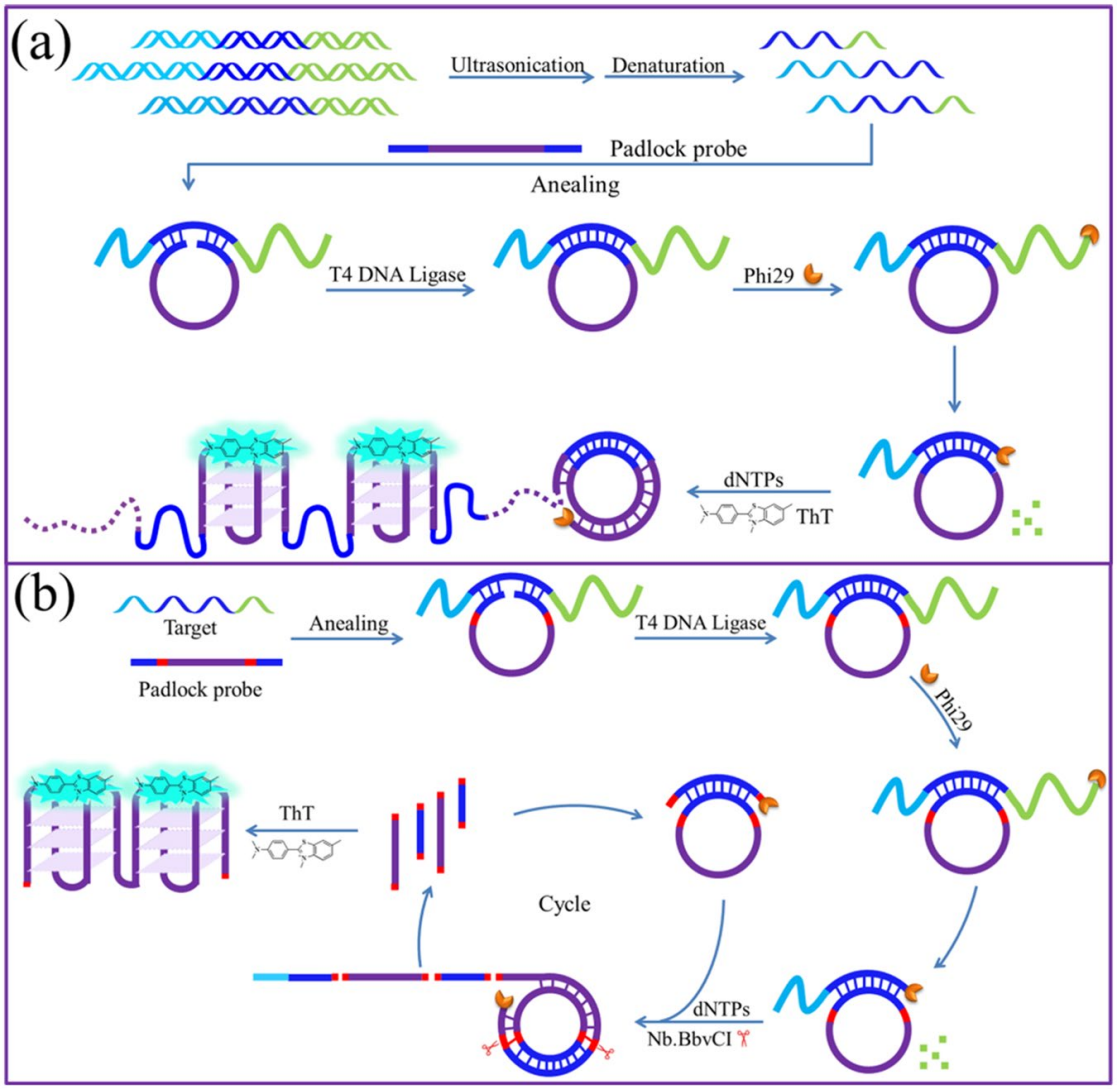
Working mechanism of ED-RCA for detection of target DNA embedded long-stranded genome. (**a**) Linear RCA mode; (**b**) Exponential RCA mode.

Our aim was to achieve RCA-amplified detection of target DNA embedded in long-stranded genomic DNA. As shown in Fig. 1a, to make the long-stranded genomic DNA fulfill the needs for RCA-based detection, we selected to cut long genomic DNA into short-stranded DNA fragments by ultrasonication. Because the resulting short DNA fragments have much simpler self-folding structures than long genomic DNA, the linear padlock probe can easily access the target DNA sequence embedded in them and be ligated by T4 DNA ligase to form a circular padlock probe, which can be used as the template in subsequent RCA. To allow the RCA reaction to proceed without the requirement of an additional RCA primer, it is better that the target DNA can be used as the primer to initiate RCA. However, due to the uncertainty of the DNA breakage sites during ultrasonication, it is possible that the target DNA sequence is still embedded in the resulting short DNA fragments. That is, different lengths of tails, which are non-complementary with the circular padlock probe, might protrude from the 5′- and 3′-ends of the target DNA sequence. Thanks to the 3′ → 5′ exonuclease activity of Phi29 DNA polymerase, the 3′-end tail can be gradually digested until the remaining part can be converted to RCA primer and is extended along the circular template via the polymerase activity of Phi29 DNA polymerase. RCA can then proceed smoothly. Herein, Phi29 DNA polymerase plays two important roles: an exonuclease and a polymerase. Although Phi29 DNA polymerase is commonly used in RCA reactions, its exonuclease activity is rarely utilized. In the proposed strategy, exonuclease activity of Phi29 DNA polymerase was used to digest the 3′-tail of target DNA to prepare the RCA primer. As a result, we named the RCA reaction an exonucleolytic digestion-assisted RCA (ED-RCA). Certainly, amplified RCA products can be determined by gel electrophoresis. However, to achieve rapid, sensitive and label-free detection, a C-rich sequence, whose complementary sequence can fold into a unique DNA secondary structure‒G-quadruplex, was introduced in the padlock probe. As the ED-RCA progresses, more and more G-quadruplexes are accumulated, which can be easily probed by the commercially available fluorescent dye thioflavin T (ThT), a highly sensitive G-quadruplex fluorescent probe showing excellent specificity against other structural DNAs, including single- and double-stranded DNAs^41–43^.

To demonstrate the feasibility of the proposed ED-RCA, several artificial DNA oligonucleotides (Table S1) with different lengths were designed to mimic the short DNA fragments obtained by ultrasonication of long genomic DNA. Each of them contains the target DNA sequence but with different tail lengths at its 3′- or/and 5′-ends. For examples, TD represents the target DNA with no tails at two ends. In 5-TD-20, a 5-nucleotide tail and a 20-nucleotide tail are added at the 5′- and 3′-ends, respectively. Other oligonucleotides are named in the same way.

**Figure 2:**
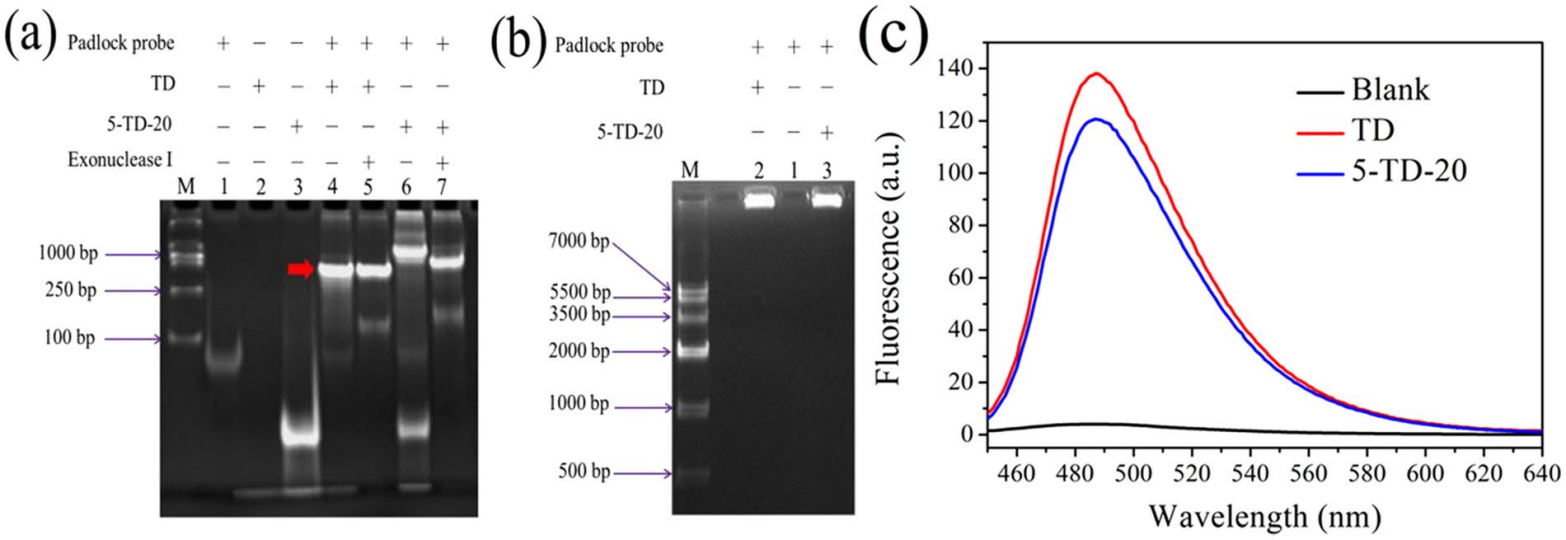
(**a**) Non-denaturing PAGE assay of circularization products produced under different conditions. Lane M is the DNA ladder marker. The experimental conditions for lanes 1–7 are shown in the top of the figure. (**b**) Agarose gel electrophoresis and (**c**) fluorescence analysis of the ED-RCA systems triggered by TD or 5-TD-20.

First, we used 5-TD-20 as a model to investigate whether the target DNA embedded in it can be detected by the proposed ED-RCA strategy or not. The results shown in Fig. 2a indicate that both TD and 5-TD-20 could trigger the circularization of a linear padlock probe. However, since the tails protruding from the two ends of 5-TD-20 increase the steric hindrance for its contact with linear padlock probe, 5-TD-20-triggered circularization had lower efficiency than the TD-triggered one. This was reflected by the presence of more unreacted single-stranded DNAs, which could be digested upon addition of exonuclease I (ExoI) in the 5-TD-20/padlock probe mixture. Interestingly, the circularization product triggered by TD had a similar electrophoretic mobility before and after digestion with ExoI. On the contrary, after ExoI digestion, the 5-TD-20-triggered circularization product showed a slightly faster mobility. These results suggest that the circularization products exist as the complexes of TD (or 5-TD-20) and padlock probe. TD could perfectly hybridize with the padlock probe, ExoI digestion was ineffective. In 5-TD-20/padlock probe complex, however, the single-stranded 3′-tail could be digested by the 3′ → 5′ exonuclease activity of ExoI, thus resulting in a slight increase in electrophoretic mobility.

From the above experiments, we find that 5-TD-20 can also trigger the formation of circular RCA template and its protruding 3′-tail can be cut off by ExoI. Thus, it is reasonable to speculate that the 3′ → 5′ exonuclease activity of Phi29 DNA polymerase might also digest the 3′-tail of 5-TD-20 and convert it to an RCA primer. As a result, the subsequent RCA reaction might be initiated without adding an additional RCA primer. Such a speculation was demonstrated by the similar results given by TD and 5-TD-20-triggered RCA systems in gel electrophoresis and fluorescence assays (Fig. 2b and c). That is, both reaction systems gave a bright electrophoresis band with a very slow mobility, indicating the formation of long-stranded RCA products. As expected, the obtained RCA products could fold into a large number of G-quadruplex units (Figure S1), thus leading to the obvious fluorescence enhancement of the G-quadruplex probe ThT. These results suggest that the proposed ED-RCA strategy might be used for the amplified detection of target DNAs embedded in DNA fragments.

**Figure 3:**
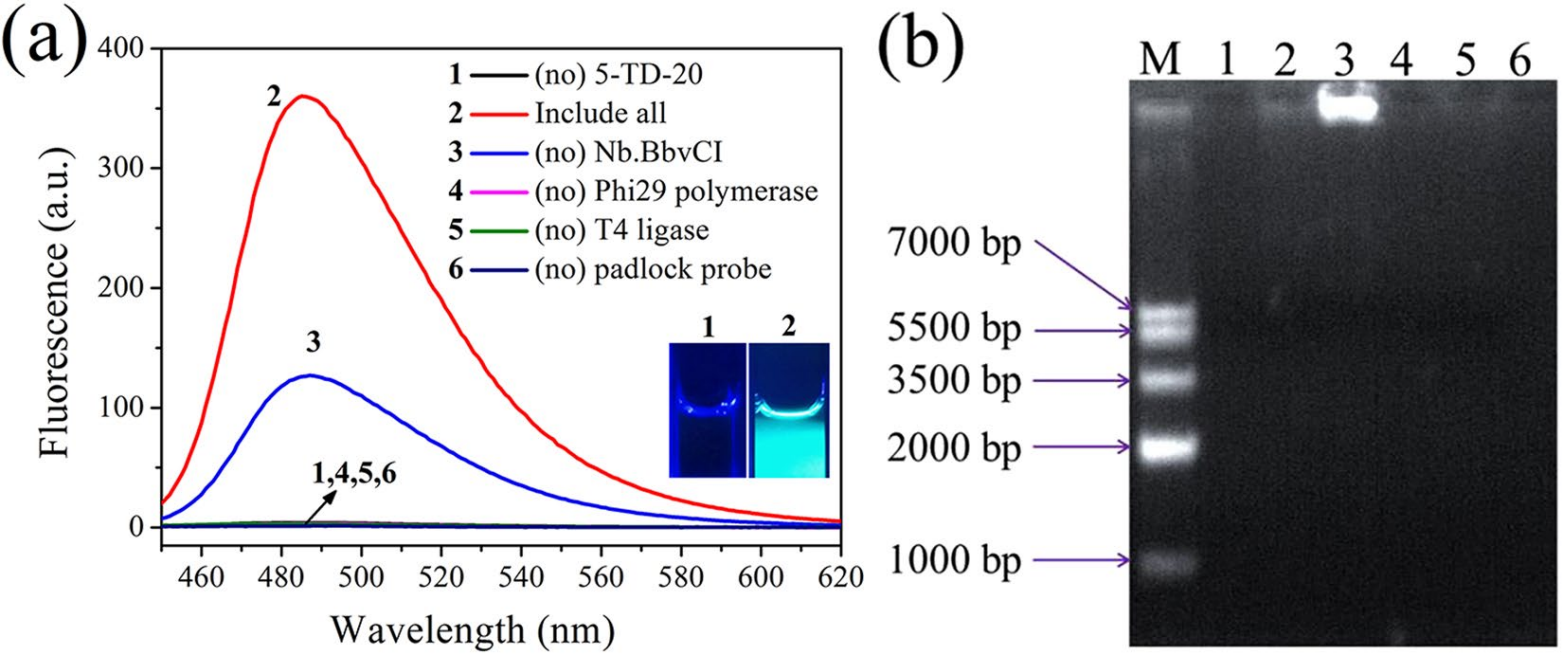
(**a**) Fluorescence and (**b**) agarose gel electrophoresis assay of 5-TD-20-triggered ED-RCA reaction systems. 1 nM of 5-TD-20 were used in fluorescence and electrophoresis assays. The insert in (**a**) shows the photographed images of the exponential RCA systems without or with 5-TD-20 addition.

A traditional RCA reaction follows a linear amplification manner with a relatively limited efficiency. To further increase the detection sensitivity, a nicking endonuclease-mediated exponential RCA strategy can be introduced (Fig. 1b)^26, 44, 45^. To achieve this, two recognition sites of a nicking endonuclease (Nb.BbvCI) were inserted in the padlock probe sequence. When the aforementioned ED-RCA reaction is initiated, the produced RCA products can be recognized and cleaved by Nb.BbvCI at the recognition sites. The resulting DNA fragments containing target DNA sequence can, in turn, hybridize with unreacted linear padlock probe and trigger the formation of new circular RCA templates. As a result, the RCA reaction is continuously enlarged and exponential signal amplification can be achieved. As shown in Fig. 3a, such an exponential ED-RCA (named ED-eRCA) indeed gave a much higher fluorescence signal than corresponding linear ED-RCA without Nb.BbvCI mediation. A gel electrophoresis assay (Fig. 3b) showed that linear ED-RCA gave a bright DNA band that corresponds to long-stranded RCA products. However, almost no observable DNA bands were given by ED-eRCA. This is consistent with the proposed mechanism that RCA products have been nicked into DNA fragments with different lengths^32^. Under the same conditions, all of the negative controls, in which one of the necessary RCA components (5-TD-20, padlock probe, Phi29 polymerase or T4 ligase) is absent, emitted no fluorescence. Such low background signals are a benefit of the excellent recognition specificity of ThT to G-quadruplexes. As long as RCA templates, primers, undesired amplification products and other components cannot fold into G-quadruplex structures, a non-specific fluorescent signal will not be given.

### Effects of protruding tails on ED-eRCA

The above experiments imply that the proposed ED-eRCA strategy has the potential to detect target DNAs embedded in DNA fragments obtained by the ultrasonication of long-stranded genomic DNA. Due to the uncertainty of DNA breakage sites during ultrasonication, different lengths of tails may occur at the two ends of the target DNA sequence. Therefore, the effects of tail length and tail position on target DNA detection were investigated.

**Figure 4:**
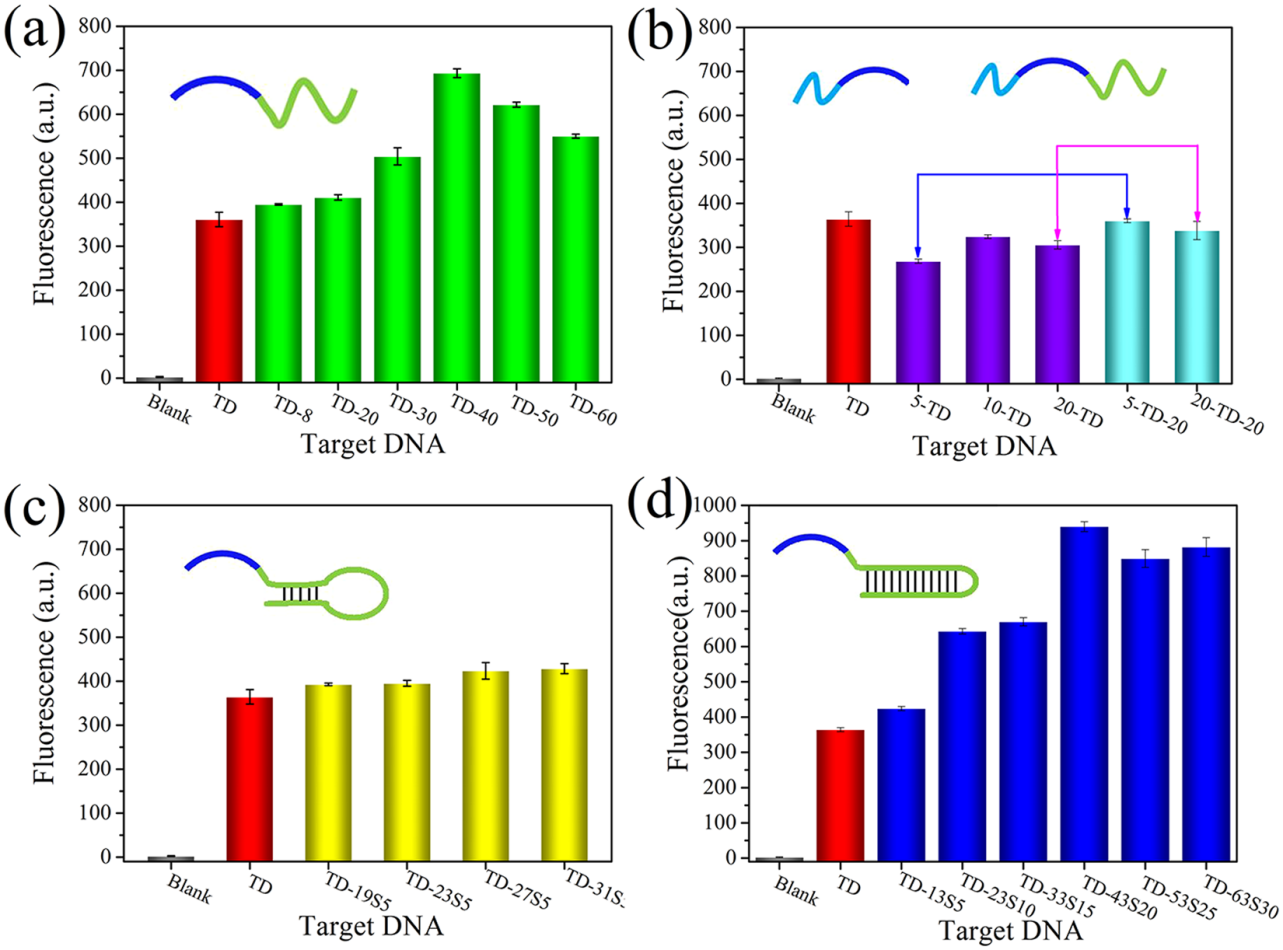
ED-eRCA analysis for different target DNA-containing fragments. (**a**) With different lengths of 3′-tails; (**b**) with different lengths of 5′-tails or both 5′- and 3′-tails; (**c**) with different lengths of 3′-tails that can fold into intramolecular stem-loop structure with 5-bp stem; (**d**) with different lengths of 3′-tails that can fold into different lengths of double-stranded structures. The concentration of each target DNA is 1 nM.

Theoretically, the 3′-tail will have much more effect on the conversion of the target DNA-containing fragment to RCA primer than the 5′-tail. Therefore, the effects of the 3′-tail were investigated first. According to our previous speculation, increasing the length of the 3′-tail should decrease the fluorescence signal of the detection system because it may hamper not only the formation of a circular template but also the conversion of the target DNA to RCA primer. Contrary to our expectation, however, increasing the length of the 3′-tail resulted in an obvious enhancement of the fluorescence signal (Fig. 4a). When the 3′-tail length was increased to 40 nucleotides (TD-40), a 1.9-fold fluorescence signal was observed compared to the TD. A gel electrophoresis assay demonstrated that increasing the 3′-tail length indeed hampered the formation of a circular template and resulted in the presence of more unreacted single-stranded DNAs (Figure S2a). Thus, the enhancement of the fluorescence signal might be caused by the improvement of target DNA conversion to RCA primer. It is reported that the 3′ → 5′ exonuclease activity of Phi29 DNA polymerase could degrade RCA primers, thus resulting in a reduction of amplification yields. To overcome this, Dean *et al*. prepared exonuclease-resistant primers by using thiophosphate linkages for the two 3′-terminal nucleotides^46^. Our results suggest that the presence of a 3′-tail can also protect RCA primers from degradation by Phi29 DNA polymerase and improve the amplification yields, and such a protection increases with tail length. This finding might provide a simple but promising way to increase RCA yields and thus improve the detection sensitivity of RCA-based sensing platforms. In our proposed ED-eRCA strategy, target DNA plays two important roles: triggering the formation of the RCA template and initiating the subsequent RCA reaction. As mentioned above, a long 3′-tail does not benefit the preparation of the RCA template. As a result, when the 3′-tail was longer than 40 nucleotides, a slight decrease in fluorescence signal was observed, but the signal level was still much higher than that given by the TD.

The effects of the 5′-tail were then investigated. Differently from the case of the 3′-tail, the fluorescence signal decrease was caused by the 5′-tail (Fig. 4b). The reason is that the presence of the 5′-tail will affect the circularization of the linear padlock probe, but cannot protect the RCA primer from degradation by Phi29 DNA polymerase. When the 3′-tail was added, the DNA fragments obtained with both 5′- and 3′-tails gave higher fluorescence signals than their counterparts with only a 5′-tail, thus demonstrating the protection of the RCA primer by the 3′-tail.

According to previous reports^39^, it seems that the 3′ → 5′ exonuclease activity of Phi29 DNA polymerase can work on single-stranded DNA but not on double-stranded DNA. In practical applications, however, it is possible that the 3′-tail may self-fold into intramolecular stem-loop structures. If Phi29 DNA polymerase cannot work on the double-stranded stem, target DNA cannot convert to the RCA primer and the RCA reaction cannot be initiated. To test this, the 3′-tail was designed as a stem-loop structure. To our surprise, the resulting DNA oligonucleotides (for example TD-31S5, which has a 31-nucleotide 3′-tail that can fold into a stem-loop structure with a 5-bp stem) could also trigger the RCA reaction, and the fluorescence signals given were still higher than that given by TD (Fig. 4c). These results indicate that the formation of a stem-loop structure at the 3′-tail will not stop the conversion of target DNA to the RCA primer, thus suggesting that the 3′ → 5′ exonuclease activity of Phi29 DNA polymerase might also be able to work for stem-loop structure. Such a speculation could be demonstrated by a gel electrophoresis assay. After incubating with Phi29 DNA polymerase, TD-31S5 could also be digested by the 3′ → 5′ exonuclease activity of this polymerase, though the digestion efficiency was lower than those of 5-TD-30 and TD-60 (Figure S2b). One possible reason is that the 3′ → 5′ exonuclease activity of Phi29 DNA polymerase might also work for double-stranded DNA, the other possible reason is that the transient dissociation of the double-stranded stem provides Phi29 DNA polymerase with an appropriate substrate. To further investigate this, 3′-tails with different lengths of double-stranded structures were added to the 3′-end of the target DNA sequence and the resulting oligonucleotides were used in the ED-eRCA assay. As shown in Fig. 4d, an obvious fluorescence enhancement was also observed with the increase of the 3′-tail length even if an increasing length of double-stranded structure was formed. For example, more than 2.6-fold fluorescence signal was observed for TD-43S20, which has a 43-nucleotide 3′-tail that can fold into a double-stranded structure with 23 base pairs, compared to TD. Moreover, the fluorescence enhancement was even higher than that give by the oligonucleotide with a similar 3′-tail length but without double-stranded structure formation (Fig. 4a). The reason might be that the formation of double-stranded structures increases the rigidity of the 3′-tail, thus reducing the steric hindrance of the hybridization between target DNA sequence and the linear padlock probe. The fact that significant fluorescence enhancement could still be observed even when the double-stranded structure was increased to 30 base pairs might preclude the possibility of a transient dissociation of the double-stranded structure. That is to say, the 3′ → 5′ exonuclease activity of Phi29 DNA polymerase is valid for both single- and double-stranded DNA. This finding greatly increases the possibility of the proposed ED-eRCA strategy for practical applications. This finding might also be useful for the future design of RCA strategies. That is, to reduce the effect of Phi29 DNA polymerase-triggered primer degradation on amplification efficiency, a primer with a well-designed 3′-tail could be used. To achieve an optimal performance, the Phi29 DNA polymerase concentration should be carefully selected to equilibrate its exonuclease activity and polymerase activity according to different demands.

### Sensitivity of ED-eRCA for target DNA detection

**Figure 5:**
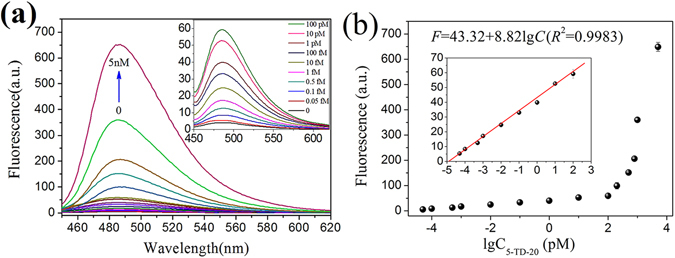
5-TD-20 quantitation using ED-eRCA. (**a**) 5-TD-20 concentration-dependent fluorescent spectral change of the detection system; (**b**) 5-TD-20 concentration-dependent fluorescent signal change at 485 nm. The insert in (**a**) shows the fluorescence spectral change in the 5-TD-20 concentration range of 0–100 pM; The insert in (**b**) shows the fluorescence signal change at 485 nm in the 5-TD-20 concentration range of 0.05 fM–100 pM. The solid line represents a linear fit to the data. All experiments were performed in triplicate.

The aforementioned experiments demonstrate that the proposed ED-eRCA strategy can be used for the detection of target DNA embedded in DNA fragments. Using 5-TD-20 as the model DNA fragment, the sensitivity of the ED-eRCA method was evaluated. As shown in Fig. 5, the fluorescence signal of the detection system continuously increased with 5-TD-20 concentration. On the logarithmic scale, a linear relationship (*R* ^2^ = 0.9983) was observed between the fluorescence signal and 5-TD-20 concentration in the range of 0.05 fM–100 pM. The linear regression equation was F = 43.32 + 8.82 lgC (pM) and the detection limit was estimated to be 0.02 fM (3σ/S), which was much lower than that given by ED-RCA with a linear amplification manner (1.48 pM, Figure S3). In the same way, DNA concentration-dependent fluorescence responses were also studied for three other DNA fragments and their mixture (Figures S4–S7). Interestingly, all of the four DNA fragments and their mixture could be quantitated in similar linear detection ranges, and no great difference was observed among the obtained linear regression equations (Figure S8 and Table S2). These results reveal that the proposed method can be used for the quantitation of the mixture of different lengths of DNA fragments containing target DNA sequence.

### SNP detection

**Figure 6:**
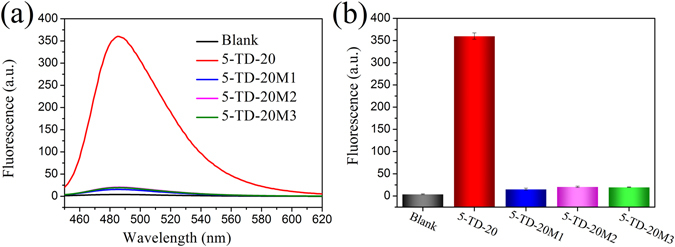
ED-eRCA-based SNPs detection. (**a**) Fluorescence spectra of the detection systems containing 5-TD-20 or its individual single-nucleotide mutants. (**b**) Corresponding fluorescence signals at 485 nm. [5-TD-20] = [5-TD-20M1] = [5-TD-20M2] = [5-TD-20M3] = 1 nM. All experiments were performed in triplicate.

SNPs are single-nucleotide variations in gene sequences. As the most abundant form of genetic variation, SNPs are the genetic basis of some diseases and are associated with drug resistance. SNP identification is crucial for not only genetic basis elucidation of complex human diseases but also the development of personalized medicine. To determine whether the proposed method could be used to identify SNPs or not, a C base in the middle of the target DNA sequence was replaced by G, A or T to generate three 5-TD-20 mutants. When these mutants were used, a mismatched base pair occurs at the ligation site, thus the linear padlock probe cannot be ligated to form a circular RCA template and the subsequent RCA reaction cannot be initiated. As shown in Fig. 6, only perfectly matched 5-TD-20 could give strong fluorescence enhancement, none of the three mutants could lead to an obvious fluorescence change compared to the blank control. Overall, target DNA-triggered circularization of the padlock probe confers the detection system with extraordinarily high specificity^47^, allowing the proposed method to easily identify SNPs embedded in long genomic DNA.

### Application to the detection of human pathogen genes

**Figure 7:**
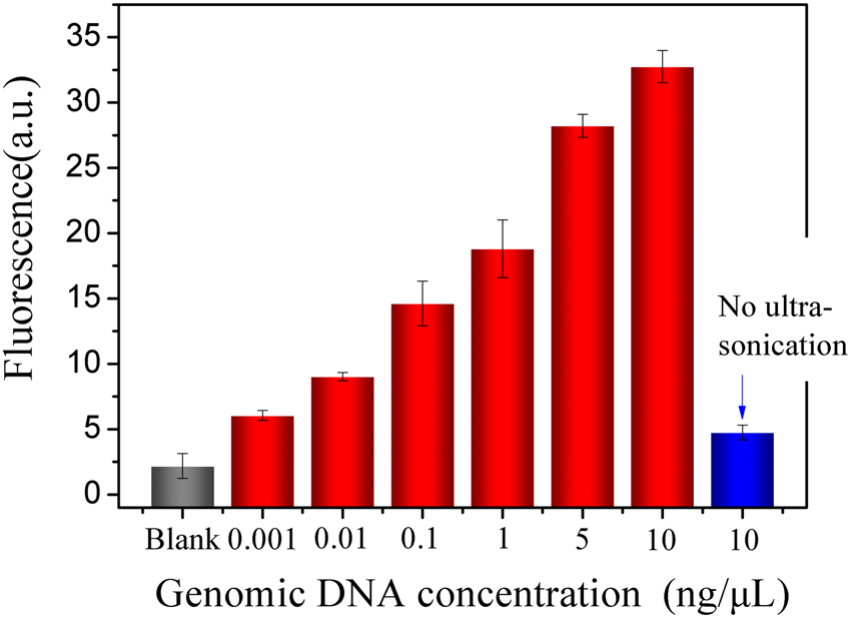
Detection of *C*. *neoformans* genomic DNA by ED-eRCA.

Finally, to evaluate the feasibility of the ED-eRCA strategy in practical applications, genomic DNA extracted from *C. neoformans* was detected. As a basidiomycete fungus, *C. neoformans* is an opportunistic human pathogen that primarily infects immunocompromised individuals, such as individuals with AIDS, organ transplant recipients and patients receiving high doses of corticosteroid treatment^48–50^. The major clinical manifestation of cryptococcosis is life-threatening meningoencephalitis, which is one of the most important HIV-related opportunistic infections, especially in the developing world^51^. Herein, to achieve the detection of *C. neoformans*, a padlock probe targeting a 23-nucleotide sequence in its virulence gene was designed (Table S3). When the pathogen genomic DNA was fragmented by ultrasonication, the padlock probe could hybridize with the DNA fragments containing target DNA sequence and be ligated by T4 DNA ligase to form a circular RCA template. Although different target DNA-containing fragments might contain different lengths of 3′-tails, all of them may be converted to RCA primer to initiate the RCA reaction since all the 3′-tails would be cut off by the 3′ → 5′ exonuclease activity of Phi29 DNA polymerase. As expected, *C. neoformans* genomic DNA concentration-dependent fluorescence enhancement was given by the detection system and the lowest detection concentration was 0.001 ng/μL (the﻿ red columns in Fig. 7). On the contrary, there is nearly no signal without ultrasonication (the blue column in Fig. 7). These results strongly demonstrate the feasibility of the proposed ED-eRCA method for the detection of target DNA embedded in long genomic DNA.

## Conclusion

In summary, by utilizing the dual functions of Phi29 DNA polymerase, 3′ → 5′ exonuclease activity and polymerase activity, an ED-eRCA method was developed for the detection of target DNA embedded in long genomic DNA. In this method, long genomic DNA is cut into small DNA fragments by ultrasonication. The target DNA-containing fragments play two roles: triggering the formation of circular RCA template and initiating the subsequent RCA reaction. Multiple functions of reaction components, combined with the greatly enhanced amplification efficiency of exponential RCA and label-free but highly specific detection of amplification products, confer the proposed ED-eRCA with the ability of highly sensitive and sequence-specific detection of target DNA embedded in different lengths of DNA fragments. Its feasibility for target DNA detection in long genomic DNA was demonstrated by using human pathogen *C. neoformans* as a model. This method was also demonstrated to be applicable for highly specific identification of SNPs occurring in long DNA fragments. This method has great application potential in a variety of areas such as environmental monitoring, food safety control, and clinical diagnosis. As byproducts, another two important discoveries were reported in this work. One is that the presence of a 3′-tail can protect the RCA primer from degradation by Phi29 DNA polymerase, thus can help to improve the amplification yields. The other is that 3′ → 5′ exonuclease activity of Phi29 DNA polymerase can work for both single- and double-stranded DNA. These findings might be useful in further improving the sensitivity of RCA-based sensing platforms and to find new applications for Phi29 DNA polymerase.

## Methods

### Materials and reagents

All oligonucleotides (Table S1) were purchased from Sangon Biotech. Co. Ltd. (Shanghai, China). Their concentrations were represented as single-stranded concentrations, which were calculated by measuring the UV absorbance at 260 nm. Molar extinction coefficient was determined by OligoAnayzer 3.1 software provided in the following website (http://sg.idtdna.com/calc/analyzer). Thioflavin T (3,6-dimethyl-2-(4-dimethylaminophenyl)benzo-thiazolium cation, ThT) was obtained from Sigma-Aldrich (Shanghai, China). T4 DNA ligase, Phi29 DNA polymerase, nicking endonuclease Nb.BbvCI, deoxyribonucleoside 5′-triphosphate mixture (dNTPs) and exonuclease I (Exo I) were obtained from New England Biolabs (Beijing, China). Agarose, ethidium bromide (EB), ammonium persulfate, DNA Marker IV and D2000 were obtained from Tiangen Biotech. Co. Ltd. (Beijing, China). N,N,N′,N′-tetramethylethylenediamine (TEMED) was obtained from Beyotime (Shanghai, China). 30% Acr-Bis (29:1) was obtained CWBIO (Beijing, China). Loading buffer (0.25% of bromophenol blue, 0.25% of xylene cyanol, 30% of glycerol and 10 mM of EDTA) and DNA ladder marker were obtained from Takara (Dalian, China). All chemical reagents were of analytical grade and used without further purification.

### Exonucleolytic digestion-triggered exponential rolling circle amplification (ED-eRCA)

Circular RCA template was prepared in 20 μL reaction mixture containing 1 × T4 DNA ligase buffer (50 mM Tris-HCl, 10 mM MgCl_2_, 1 mM ATP, pH 7.5), 500 nM linear padlock probe and different concentrations of target DNA-containing oligonucleotide. To ensure that padlock probe can fully hybridize with target DNA, the mixture was heated at 95 °C for 5 min, and then cooled to 37 °C and incubated at this temperature for 0.5 h. After addition of 20 U of T4 DNA ligase, the mixture was allowed to incubate at 16 °C for 4 h to ensure that the 5′-phosphate and 3′-hydroxyl ends of linear padlock probe can be ligated to form a circular template. Above mixture was prepared in 1 × Phi29 DNA polymerase buffer (50 mM Tris-HCl, 10 mM (NH_4_)_2_SO_4_, 4 mM DTT, pH 7.5), 3 µg/mL of BSA, 250 μM each dNTP, 5 U Phi29 DNA polymerase, 5 U Nb.BbvCI and deionized water. The obtained 100 μL reaction mixture was incubated at 30 °C for 5 h to perform RCA reaction. Then, 8 μM ThT (final concentration) was added and sufficiently mixed. Corresponding fluorescence signal of the mixture was measured on a Shimadzu RF-5301pc fluorescence spectrometer (Shimadzu Ltd., Japan). The emission spectra were collected from 450 nm to 620 nm using 425 nm as the excitation wavelength, and the fluorescence signal intensity at 485 nm was used for quantitative analysis of target DNA-containing DNA fragments.

### Non-denaturing polyacrylamide gel electrophoresis (PAGE) analysis of RCA template production and the 3′ → 5′ exonuclease activity of Phi29 DNA polymerase

Target DNA-assisted circular RCA template formation was verified by non-denaturing PAGE analysis. 20 μL ligation reaction solution was mixed with 4 μL loading buffer. The mixture was loaded onto a 18% polyacrylamide gel, and PAGE analysis was carried out in 1 × TBE buffer (89 mM of tris-boric acid, 2.0 mM of EDTA, pH 8.3) at a constant potential of 120 V for 1 h. After staining by EB solution for 30 min, the gel was photographed by a gel documentation system (Huifuxingye, Beijing, China). The 3′ → 5′ exonuclease activity of Phi29 DNA polymerase was also demonstrated by non-denaturing PAGE analysis. Solutions containing individual target DNAs (5-TD-20, TD-60 or TD-31S5) were heated at 95 °C for 5 min, and then cooled to 37 °C and incubated at this temperature for 0.5 h. After incubating with 0.5 or 1 U/μL Phi29 DNA polymerase at 30 °C for 10 h, non-denaturing PAGE analysis was conducted as above.

### Agarose gel electrophoresis analysis of RCA products

RCA products were separated and analyzed by agarose gel electrophoresis. Similar to PAGE analysis, RCA reaction solution (15 μL) was sufficiently mixed with 3 μL loading buffer. The mixture was loaded onto a 2% agarose gel, and the electrophoresis analysis was carried out in 1 × TBE buffer at a constant potential of 70 V for 1 h. After staining by EB solution for 30 min, the gel was photographed using the gel documentation system.

### Circular dichroism (CD) spectra analysis of RCA products

CD spectral analysis was used to demonstrate the formation of G-quadruplex structure in RCA products. 3 mL RCA reaction mixture was collected and its CD spectrum was recorded between 220 and 320 nm in 1 cm path length cuvette on a Jasco J-715 spectropolarimeter at room temperature. Spectra were averaged from three scans, which were recorded at 100 nm/min with a response time of 1 s and a bandwidth of 0.5 nm.

### Pathogen genomic DNA analysis

*Cryptococcus neoformans* var. neoformans strain JEC21 (serotype D) was the generous gift of Prof. Xudong Zhu (Nankai University, Tianjin, China). Corresponding cryptococcal genomic DNA was extracted according to the protocol described by Casadevall and Perfect^52^ and quantified by measuring the UV absorbance at 260 nm. Then, the extracted genomic DNA was treated with ultrasonication at 0 °C using 40 consecutive 30s-on/30s-off cycles at low power (KQ-300 DE numerical control ultrasonic cleaners, China) to fragment the long DNA chains. The obtained short DNA fragments were diluted with Tris-HCl buffer (20 mM, pH 7.4) to different concentrations and were analyzed by the proposed ED-eRCA method as above described.

## Electronic supplementary material

Supporting information
