# Supplementary material for: Dual functional Phi29 DNA polymerase-triggered exponential rolling circle amplification for sequence-specific detection of target DNA embedded in long-stranded genomic DNA
Source: Sci Rep. 2017 Jul 24;7:6263. doi: 10.1038/s41598-017-06594-1 (PMC5524717; doi:10.1038/s41598-017-06594-1)
Supplement: Supplementary file 1 — Supporting information [file 41598_2017_6594_MOESM1_ESM.doc]

Supporting information for

**Dual functional Phi29 DNA polymerase-triggered exponential rolling circle amplification for sequence-specific detection of target DNA embedded in long-stranded genomic DNA**

Xiao-Yu Li^1,2^, Yi-Chen Du^2^, Yu-Peng Zhang^2^ and De-Ming Kong^1,2,3^*

^1^ State Key Laboratory of Medicinal Chemical Biology, Nankai University, Tianjin, 300071, P. R. China

^2^ Tianjin Key Laboratory of Biosensing and Molecular Recognition, Research Center for Analytical Sciences, Nankai University, Tianjin, 300071, P. R. China

^3^ Collaborative Innovation Center of Chemical Science and Engineering (Tianjin), Tianjin, 300071, P. R. China

* Corresponding author. Tel.: +86-22-23500938; Fax: +86-22-23502458

E-mail address: kongdem@nankai.edu.cn (D.-M. Kong).

1. **The DNA oligonucleotides used in this work**

**Table S1. The oligonucleotides used in this work**

| **Name** | **Sequence (5′→3′)** |
| --- | --- |
| **Padlock probe** | Phosphate-TGA CAT TGT A**CC TCA GC**C CTA ACC CTA ACC CTA ACC CTT ACC CTA ACC CTA ACC CTA AC**C CTC AGC** TTG AAT CCG TG |
| **TD** | TAC AAT GTC ACA CGG ATT CA |
| **TD-10** | TAC AAT GTC ACA CGG ATT CAT TCG ATT CGA |
| **TD-20** | TAC AAT GTC ACA CGG ATT CAT TCG ATT CGA TTC GAT TCG A |
| **TD-30** | TAC AAT GTC ACA CGG ATT CAT TCG ATT CGA TTC GAT TCG ATT CGA TTC GA |
| **TD-40** | TAC AAT GTC ACA CGG ATT CAT TCG ATT CGA TTC GAT TCG ATT CGA TTC GAT TCG ATT CGA |
| **TD-50** | TAC AAT GTC ACA CGG ATT CAT TCG ATT CGA TTC GAT TCG ATT CGA TTC GAT TCG ATT CGA TTC GAT TCG A |
| **TD-60** | TAC AAT GTC ACA CGG ATT CAT TCG ATT CGA TTC GAT TCG ATT CGA TTC GAT TCG ATT CGA TTC GAT TCG ATT CGA TTC GA |
| **5-TD** | AGC TTT ACA ATG TCA CAC GGA TTC A |
| **10-TD** | AGC TTA GCT TTA CAA TGT CAC ACG GAT TCA |
| **20-TD** | AGC TTA GCT TAG CTT AGC TTT ACA ATG TCA CAC GGA TTC A |
| **5-TD-20** | AGC TTT ACA ATG TCA CAC GGA TTC ATT CGA TTC GAT TCG ATT CGA |
| **20-TD-20** | AGC TTA GCT TAG CTT AGC TTT ACA ATG TCA CAC GGA TTC ATT CGA TTC GAT TCG ATT CGA |
| **TD-19S5** | TAC AAT GTC ACA CGG ATT CAA AAA AAC GTT AAA ATA ACG |
| **TD-23S5** | TAC AAT GTC ACA CGG ATT CAA AAA AAA AAA CGT TAA AAT AAC G |
| **TD-27S5** | TAC AAT GTC ACA CGG ATT CAA AAA AAA AAA AAA ACG TTA AAA TAA CG |
| **TD-31S5** | TAC AAT GTC ACA CGG ATT CAA AAA AAA AAA AAA AAA AAC GTT AAA ATA ACG |
| **TD-13S5** | TAC AAT GTC ACA CGG ATT CAT TCG AAA ATC GAA |
| **TD-23S10** | TAC AAT GTC ACA CGG ATT CAT TCG ATT CGA AAA TCG AAT CGA A |
| **TD-33S15** | TAC AAT GTC ACA CGG ATT CAT TCG ATT CGA TTC GAA AAT CGA ATC GAA TCG AA |
| **TD-43S20** | TAC AAT GTC ACA CGG ATT CAT TCG ATT CGA TTC GAT TCG AAA ATC GAA TCG AAT CGA ATC GAA |
| **TD-53S25** | TAC AAT GTC ACA CGG ATT CAT TCG ATT CGA TTC GAT TCG ATT CGA AAA TCG AAT CGA ATC GAA TCG AAT CGA A |
| **TD-63S30** | TAC AAT GTC ACA CGG ATT CAT TCG ATT CGA TTC GAT TCG ATT CGA TTC GAA AAT CGA ATC GAA TCG AAT CGA ATC GAA TCG AA |
| **5-TD-20M1** | AGC TTT ACA ATG TCA *G*AC GGA TTC ATT CGA TTC GAT TCG ATT CGA |
| **5-TD-20M2** | AGC TTT ACA ATG TCA *A*AC GGA TTC ATT CGA TTC GAT TCG ATT CGA |
| **5-TD-20M3** | AGC TTT ACA ATG TCA *T*AC GGA TTC ATT CGA TTC GAT TCG ATT CGA |

In padlock probe, the green and blue sequences are the ones that can hybridize with target DNA sequence; the red sequences are the recognition sites of nicking endonuclease Nb.BbvCI; the underlined sequence is the C-rich one whose complimentary sequence can fold into dimeric G-quadruplex structure. In 5-TD-20M1, 5-TD-20M2, 5-TD-20M3, the SNP sites are shown in italic and underlined.

1. **Diagrammatic presentation of the intramolecular double-stranded structures formed by 3′-tails**


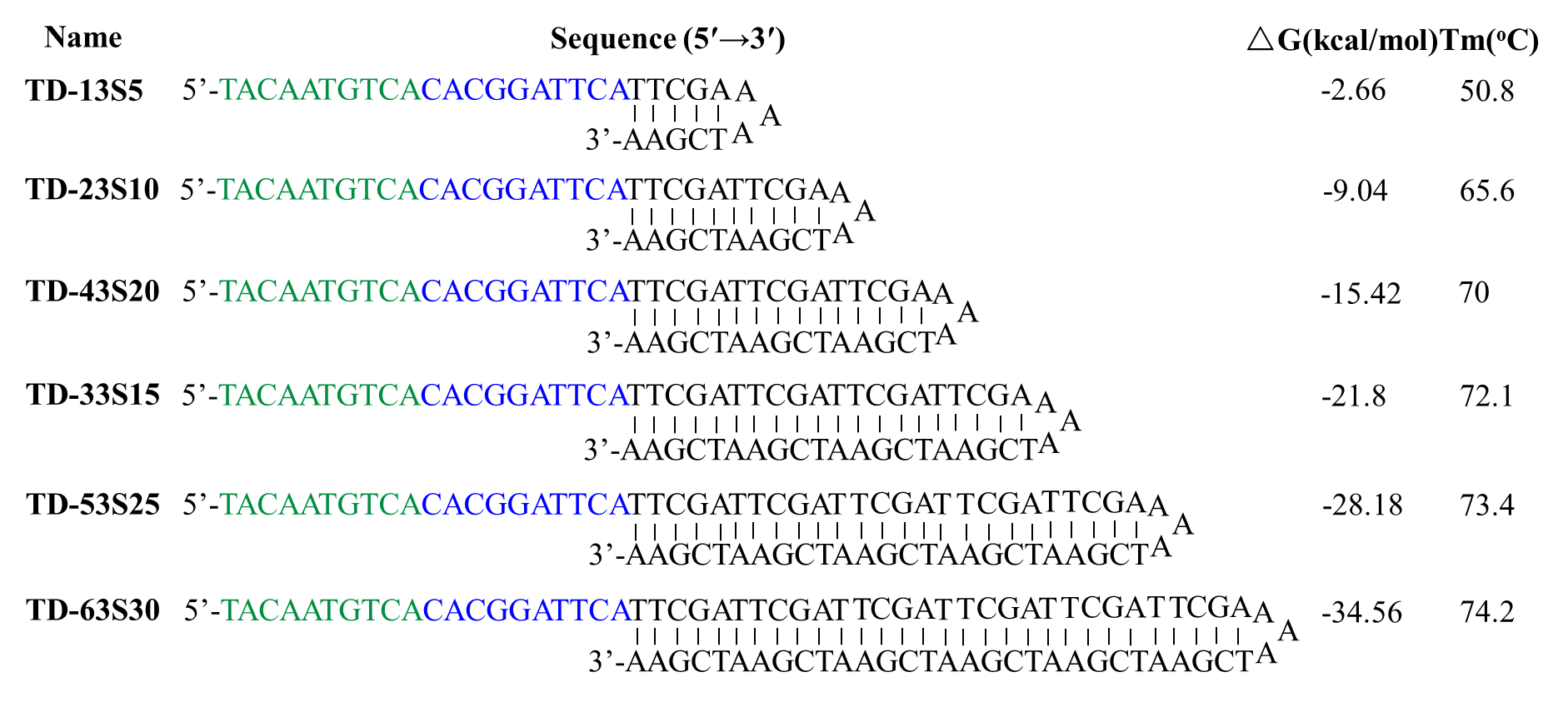


1. **Circular dichroism (CD) spectral analysis of RCA products.**

CD spectral analysis was used to demonstrate the formation of G-quadruplex structure in RCA products. As shown in Figure S1, successful RCA reactions, in which TD or 5-TD-20 was used, resulted in the emergence of a strong positive CD peak at around 288 nm (or 286 nm), a weak positive peak at around 261 nm and a negative peak at around 240 nm, indicating the formation of parallel/antiparallel hybrid G-quadruplex structure, which is the typical structure formed by human telomere G-quadruplexes embedded in presumptive RCA products. The presence of ThT showed no effects on the G-quadruplex structure.

**Figure S1.** CD spectral analysis of RCA reaction mixtures with or without target DNA. Presumptive RCA products can be produced only in the presence of target DNA (TD or 5-TD-20).

1. **Non-denaturing PAGE analysis of circular RCA template production and the 3′→5′ exonuclease activity of Phi29 DNA polymerase**


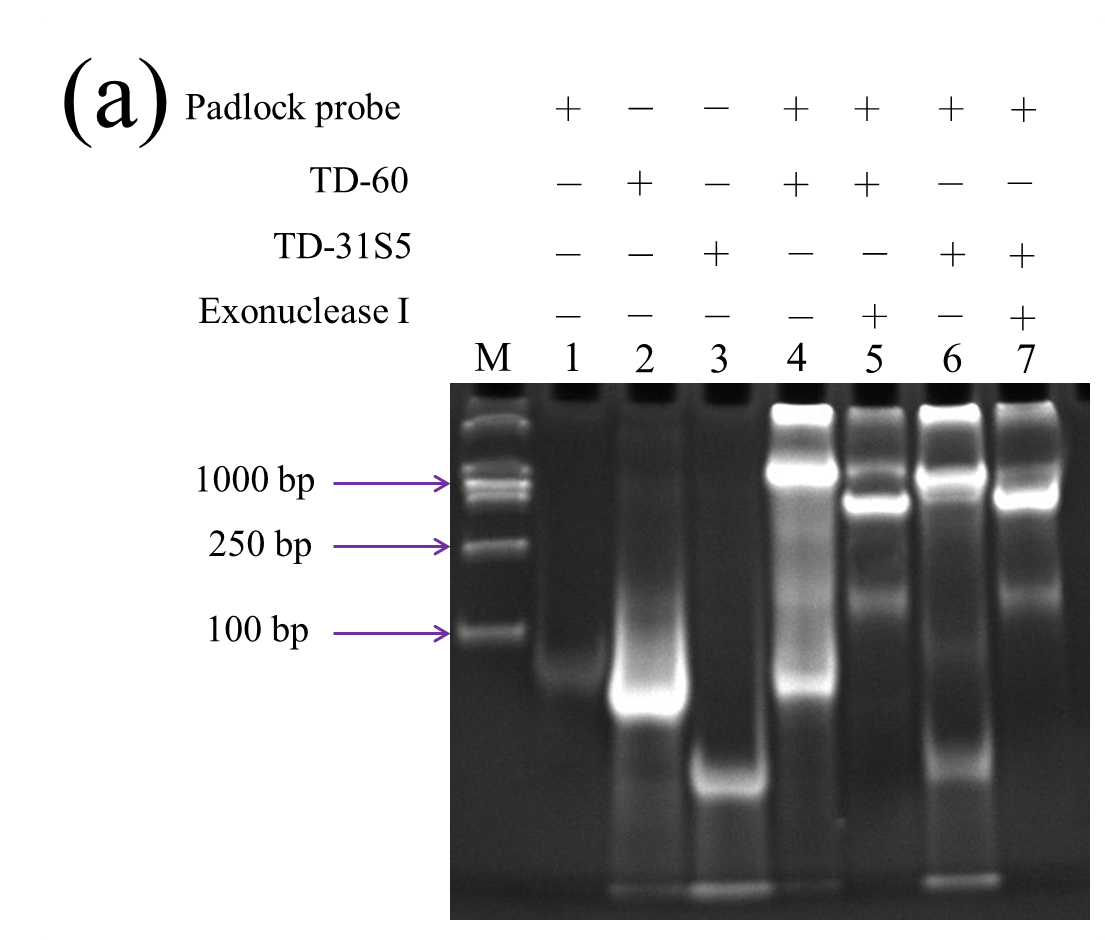

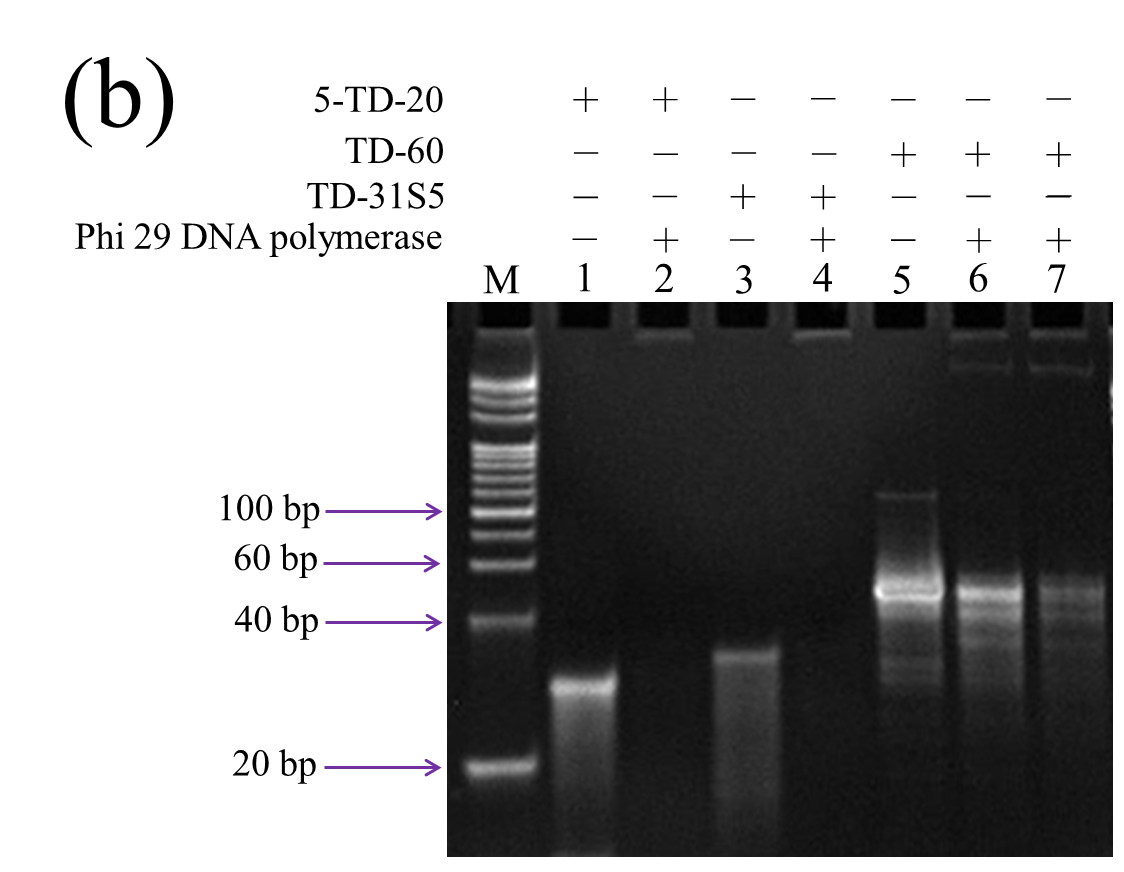


**Figure S2.** (a) Non-denaturing PAGE analysis of TD-60 or TD-31S5-triggered formation of circular RCA template. Lane M is the DNA ladder marker. The experimental conditions for lanes 1-7 are shown in the top of the figure. (b) Non-denaturing PAGE analysis of the 3′→5′ exonuclease activity of Phi29 DNA polymerase towards 5-TD-20, TD-60 and TD-31S5. Lane M is the DNA ladder marker. The experimental conditions for lanes 1-7 are shown in the top of the figure. The concentration of Phi29 DNA polymerase was 0.5 U/μL in lanes 2, 4, 6; and the concentration was 1 U/μL in lane 7.

1. **5-TD-20 quantitation using linear ED-RCA.**

To demonstrate that the exponential amplification strategy of ED-eRCA can really increase the detection sensitivity, 5-TD-20 quantitation was also conducted by linear ED-RCA without Nb.BbvCI mediation. As shown in Figure S3, a linear relationship (*R*^2^ = 0.9945) was observed between the fluorescence signal and 5-TD-20 concentration in the range of 50 pM – 200 pM. The linear regression equation was F = -0.6146 + 118.9 C (nM) and the detection limit was estimated to be 1.48 pM, which was obviously higher than that given by ED-eRCA (0.02 fM).

**Figure S3.** 5-TD-20 concentration-dependent fluorescent signal change at 485 nm. The insert shows the fluorescence signal change in the 5-TD-20 concentration range of 50 – 200 pM. The solid line represents a linear fit to the data. All experiments were performed in triplicate.

1. **Quantitation of different target DNA-containing fragments by ED-eRCA**
   1. **Quantitation of TD**

**Figure S4.** TD quantitation using ED-eRCA. (a) TD concentration-dependent fluorescent spectral change of the detection system; (b) TD concentration-dependent fluorescent signal change at 485 nm. The insert in (a) shows the fluorescence spectral change in the TD concentration range of 0 – 100 pM; The insert in (b) shows the fluorescence signal change at 485 nm in the TD concentration range of 0.1 fM – 100 pM. The solid line represents a linear fit to the data. All experiments were performed in triplicate.

- 1. **Quantitation of TD-60**

**Figure S5.** TD-60 quantitation using ED-eRCA. (a) TD-60 concentration-dependent fluorescent spectral change of the detection system; (b) TD-60 concentration-dependent fluorescent signal change at 485 nm. The insert in (a) shows the fluorescence spectral change in the TD-60 concentration range of 0 – 100 pM; The insert in (b) shows the fluorescence signal change at 485 nm in the TD-60 concentration range of 0.05 fM – 100 pM. The solid line represents a linear fit to the data. All experiments were performed in triplicate.

- 1. **Quantitation of TD-31S5**

**Figure S6.** TD-31S5 quantitation using ED-eRCA. (a) TD-31S5 concentration-dependent fluorescent spectral change of the detection system; (b) TD-31S5 concentration-dependent fluorescent signal change at 485 nm. The insert in (a) shows the fluorescence spectral change in the TD-31S5 concentration range of 0 – 100 pM; The insert in (b) shows the fluorescence signal change at 485 nm in the TD-31S5 concentration range of 0.1 fM – 100 pM. The solid line represents a linear fit to the data. All experiments were performed in triplicate.

- 1. **Quantitation of DNA mixture**

**Figure S7.** DNA mixture detection using ED-eRCA. The mixture was prepared by mixing TD, TD-60, TD-31S5 and 5-TD-20 with the concentration ratio of 1:1:1:1. (a) Total DNA concentration-dependent fluorescent spectral change of the detection system; (b) Total DNA concentration-dependent fluorescent signal change at 485 nm. The insert in (a) shows the fluorescence spectral change in the total DNA concentration range of 0 – 100 pM; The insert in (b) shows the fluorescence signal change at 485 nm in the total DNA concentration range of 0.1 fM – 100 pM. The solid line represents a linear fit to the data. All experiments were performed in triplicate.

- 1. **Comparison of linear regression equations**

**Figure S8.** Quantitation of four target DNA-containing fragments and their mixture by ED-eRCA.

**Table S2.** Linear regression equations of the tested DNA fragments and their mixture

| **DNAs** | **Linear regression equation** |
| --- | --- |
| TD | *F* = 39.76 + 8.87 lg*C* (pM) |
| TD-60 | *F* = 46.35 + 9.80 lg*C* (pM) |
| TD-31S5 | *F* = 41.81 + 8.97 lg*C* (pM) |
| 5-TD-20 | *F* = 43.32 + 8.82 lg*C* (pM) |
| Mixture of TD, TD-60, TD-31S5 and 5-TD-20 (1:1:1:1) | *F* = 39.93 + 9.35 lg*C* (pM) |

1. **Sequences of *C. neoformans* genomic DNA and corresponding padlock probe**

**Table S3. Sequences of *C. neoformans* genomic DNA and corresponding padlock probe**

| **Name** | **Sequence (5′→3′)** |
| --- | --- |
| **Padlock probe** | Phosphate-ACG ATC TTG GAC CTC AGC CCT AAC CCT AAC CCT AAC CCT TAC CCT AAC CCT AAC CCT AAC CCT CAG CTT CAC TTG ACT CG |
| **Human disease-related *C. neoformans* chromosome 7 genomic DNA** | GGGAGACGTGCGCTGCTCCACGGGAATGGGTGTACGTTAGTTTACAGCGCCCCAAAGGTCGCTGTACACTCCGTGCCCGGTCGGTTCCCACAATCTTTCGCAGGTATGACCCCTATCCGTCCTCTGCCGTATGCGCGAACTTCAGGTTCACTTATGTCGTACTGCAATCAGATGGAGATCACCGACGAGCGAACTTAAGGTTTCCATATTCAATTAGTGACAGTCAGTATCCGACGGCATAACGACTATCCTGAAATAGCGACAGTCCTTTCTTTATCAGTATGTCCCTGATTCAGTCTTCTTTCGGGAAGCGAACATGGATCACGGGCAAAGCAGCAAGCATGGACTTGGAACAGCAACCACACTACTGCCAAGGGTGCGATACATCATTCACAGCATTGTGAGCGCATGCCCGATATCAAAGGCCATCAGAGAACTTGCTTCTGACTTTGTGCTACAGCGGATATCCGGTAGGAGAGACATATATAAGCCTGGTTAACTGAAAAGGCCTCATTTTACAATCAGCTCTACATAATTGGCCTCCTGAAGAGCAACTTCTCCCTTCAAGTCCAGAGTATCAACCCTTATCGGCACTTCTGAGGCATTATACTGGTGTAATATCTTATCTTTATCCTAATCGGTAAATATTTCTTTCTCTTCTCTGTCTCGGTCCTGTTCCGCCGTCGATCGATATACGCCAGCAGCCTTTTCCCATTCTTTCATGGCTACCTGAAGCGCTCCAAGATCGTCGAGTCAAGTGACCGTACTCGTGCCGGGCCTTGCTGTCGCTACCTGATCAAAGTCTTCCATGAAAGGATGAACTGTCGCCCTCCTACACTCAGACGGTGCGACCGCTTTAGCTTCCTTGCTACTCTTTTACTGCGCTCGATACATTCTACTTTTGAATTCAACCTTCACAACTCGCATCTTCCCACTATCAACTTCAGAATCTCCATTTTGTTTCCAGCGTCCATAATACTCTATGCTAATAGGTCAATTTAGCTATACCGACGCTCACAGTAATGCGGGGGTTAGCCAAGCTTTTCTTCCTATCCTGTTCCTTCGTTTCGCTGGTCAGCAGCGAGAAGACTGATGAGTGAGTTGATGTCGTTGAGTTTCGCAAGGTCCAAGGAAGGGCTAATCACTGTTCAGGTCGCCAACCGCGGTTTCTGATAACTATATGCCGAAAGCGACAGCAACCATTGACCCTAGTGTATTCGCTCTTTCAAATGACTTTGAAATAACAGATGTTCCGACGACGAGAGAGTATACCTTCGATATCGCGTGAGTCGGAATTTAAAGATCGCGTATTTTAATCTGACGCACAATTGCAGCAACGCTTTCGCCAGCCCTGATGGTTATGAACGGGAGGTTTACGTTGTCAACAACATGTTCCCTGGTCCTGTGATAGAGGCTAACACCGGCGATACTATTATCGTACATGTCAACAATCATTTGGATGAAGGACAAAGTCTCCGTAAGCATCAGCTCATATCCTGCTCATATTCAAAGACGCACTTTTAATGTACTAACTCTTAAATCCAGACTGGCATGGTTTGCGGCAGCTGGGCACGGCTTTCATGGACGGTGTCCCTGGTATAACTCAGGTAAGAATCCCTAGAAACCACAGGAATTGGTCTTAACTGATAAATTGGTTAGTGTCCTATTCCGCCTGGAGGCTCATTTACCTACAATTTCACCGTAAGCCATCAATCCGGCACGTATTGGTGGCACTCCCATTACTCCAATTCCATGGCCGACGGCATTTGGGGCCCGTCAGTTCTCCTGACTTCTATAGCAACTTATCACAGCTGATAAGATATCATAGCCTAATTGTGCACTCGCCCAATGAACCCCTTCAGAGGGGACGAGACTATGATGAGGACCGAATCGTTTTTATAACTGACTGGATGTAAGTATGTCCGTTCTTCAATGAGACTCAGCTAAACATTGACGATGGCAGGCATGATAACTCAGAAATCATCATTGCAGCTCTAGCCACTCCAGAAGGATACAAAGGAGTGAGTAAAAAAAAGTTTAAGGGTTGATAGCCCAACACTGACTGTAAGAAAGAACATTGCTCCTCCACAAGGTGATGCGATTCTCATCAACGGCCGTGTACGTGGTTTTACTGGCAACAACGATAACCCCCACGTAGACTTACTAACACTTACCGCTAGGGCCAAACAAATTGCACAGCTACTGGTTCCTCGTCATGCTTCTATCCCCCACCTCCGGAGATCCAAGTGCCCGTCAACTGCAGGGTTCGTCTGCGCTTTATCAGCGCGACCGCCCATCCCATGTACCGCATATCTATCGACAACCACCCTATGGAAGTTGTGGAGGCCGATGGTACTGCCGTCTATGGGCCGACAGTCCATGAAATCTCCATCGCACCCGGGGAACGGTACTCTGCAATTATCAACACCAATGAAGGGAAAGAAGGTGATGCGTTTTGGCTGAGGACTAGCGTTGCTCTAAGCTGTATGTTTGGTGCAGTAAGTCAGGAGGGATTGGCGGTGGTGAGGTATACGGGTAATGGAATCGTTAGTACTGAGGAGCCTCAGACTTCTGCTTGGTGAGTTACCGAAAAATCTCAGGTTGAACTGGCGCTGACAATATATTCAGGAGTGATCTAGCGGGAGTTACCGTTCCATGTACTGGACTGGACCAAACATATACTCTTTCGTGAGCATTGGTTGATTTCTCTTGTTTGAATCTTTTTACTAATAACTACGTAGACCACGAGACAGTCTTAGTGCACCTCGGGAACCTTTGCAAAGCCATTTCTTCAATAGCGAACGAGGAGCCTTTGTGAATGTTCTTGGTAATACCTTCCAGGGTTATGTATGTAATTTTCCATTTTCCAGTATTACATTCAAGGGAAAACGTGCTGACAGTTATCTAGGGGTTCAACAATATCTCATATCAGAACCAAATCTTCAACCCTCTACTTTCAATCGTCCAACGCGGAGGCTCCTGCGAGAACACATTGGTATCCAGTAGAACTTTCCCCGACTTCGGGCCAGGGAACATTATCATCAACAATCTTGATACCGTCATCGACCATCCTTACCACCTGCACGGCAACGAGTTCCAGGTGATAGGACGAGGTACTGGAGCTCTCAGTATTGATAACCTGACTAATATTGACTTCACTTTGGACAACCCCGTGAGAAAGGATACCCTCTGGATACAGGGTGGAAGTTGGGCGGTATTGAGGATCACAGCGGATAACCCTGGAGTTTGGGCCTTGCATTGTCATATTGGGTGGCATCTTACTGAGGGAAAGGTGAGTTTTTATCCTTCGTTCGCCCATTCACCCACTAAAATTGTTTATCTAGTTGGCTGTGATTGTCGTTCAACCAAGTGCGATTGGACATATGGAGAGCCCCGAGTCTTGGACGAATGTGAATCGAGCTTCTTATTTTCAAAGAGCTTTTGCTAACTTATTTCTCCCTTTAGCTCTGTGCTAACACCGATCCCAATGCGTTTGGCCCGGCAAAACGCTCATCTTCTCCGTCTATTCAATCCTCTAAGACATCCAGTTTCCAGTATCTCCGTGAAGTCAAAGGGAAGGTTGTTAAACGTAGAGGTGCGCGAGAGGCGTGAGAGACCGCTCGGGTTGGAAGAATCATTGGCCAATGATATGAAGAAGAATTGGGGGGTAATGCGGCTTGGTACGAGAAGGGGTATCACAGGCCACTCTATCTGACGGACTTTTTGGTTAACTGGGGGTTTTATATTATTCCTTCAATGGACTTTATCAAAGAATGCTCATCTAATCGTGTACCTATAGCAAACAATACTTTCTTTTACGATAATGCCATCAAAAATTCGATAGCATCATTTCAGTCACTCTGATGTATTTATG |

In *C. neoformans* var. *neoformans* JEC21 chromosome 7 genomic DNA, the underlined blue sequence is the target DNA sequence probed by padlock probe.
